# Retraction Note: Chemical inhibition reveals differential requirements of signaling pathways in *krasV12*- and *Myc-*induced liver tumors in transgenic zebrafish

**DOI:** 10.1038/s41598-023-48297-w

**Published:** 2023-11-29

**Authors:** Chuan Yan, Qiqi Yang, Xiaojing Huo, Hankun Li, Li Zhou, Zhiyuan Gong

**Affiliations:** 1 Department of Biological Sciences, National University of Singapore, Singapore, Singapore; 2 National University of Singapore graduate school for integrative sciences and engineering, National University of Singapore, Singapore, Singapore

Retraction of: *Scientific Reports* 10.1038/srep45796, published online 05 April 2017

The Editors have retracted this Article.

After publication of this Article, the Authors discovered that some of the data in the paper is duplicated. Specifically, the following panels are identical:

- Figures 1C and 3A,
- Figures 1G and 3H,
- Figures 2E and 3F, and,
- Figure 5G and 5K.

Since the original data for these panels is no longer available, the Editors decided to retract this Article.

Chuan Yan, Qiqi Yang, and Zhiyuan Gong agreed to the retraction. Xiaojing Huo and Hankun Li did not respond to the correspondence about this retraction. The Editors were not able to establish the current contact details for Li Zhou.

